# The Human Cytomegalovirus US31 Gene Predicts Favorable Survival and Regulates the Tumor Microenvironment in Gastric Cancer

**DOI:** 10.3389/fonc.2021.614925

**Published:** 2021-04-20

**Authors:** Sisi Ye, Yuanbo Hu, Chenbin Chen, Sian Chen, Xinya Tong, Huanbo Zhu, Bo Deng, Xianjing Hu, Xiangwei Sun, Xiaodong Chen, Xinyu Shi, Ruihong Gu, Wangkai Xie, Gangqiang Guo, Dong Xing, Xian Shen, Xiangyang Xue, Shurong Shen

**Affiliations:** ^1^ Department of Medical Microbiology and Immunology, Wenzhou Medical University, Wenzhou, China; ^2^ Department of Precision Medical Center Laboratory, The First Affiliated Hospital of Wenzhou Medical University, Wenzhou, China; ^3^ Department of Gastrointestinal Surgery, Second Affiliated Hospital and Yuying Children’s Hospital of Wenzhou Medical University, Wenzhou, China; ^4^ Wenzhou Hospital of Integrated Traditional Chinese and Western Medicine, Zhejiang Chinese Medical University, Wenzhou, China

**Keywords:** human cytomegalovirus, gastric cancer, US31, tumor immune microenvironment, prognostic factor

## Abstract

Human cytomegalovirus (HCMV) is an oncogenic virus associated with tumorigenesis. Our previous study revealed that the HCMV US31 gene interacted with NF-κB2 and mediated inflammation through macrophages. However, there are few reports on the role of US31 in gastric cancer (GC). The aim of this study was to investigate the expression of the US31 gene in GC tissue and assess its role in the occurrence and development of GC. US31 expression in 573 cancer tissues was analyzed using immunohistochemistry. Results showed that US31 was significantly associated with tumor size (*P* = 0.005) and distant metastasis (*P* < 0.001). Higher US31 expression indicated better overall survival in GC patients. Overexpression of US31 significantly inhibited the proliferation, migration, and invasion of GC cells *in vitro* (*P* < 0.05). Furthermore, expression levels of CD4, CD66b, and CD166 were positively correlated with US31, suggesting that it was involved in regulating the tumor immune microenvironment of GC. RNA sequencing, along with quantitative real-time polymerase chain reaction, confirmed that the expression of US31 promoted immune activation and secretion of inflammatory cytokines. Overall, US31 inhibited the malignant phenotype and regulated tumor immune cell infiltration in GC; these results suggest that US31 could be a potential prognostic factor for GC and may open the door for a new immunotherapy strategy.

## Introduction

Gastric cancer (GC) is the fourth most common malignant tumor and second leading cause of tumor-related deaths in the world (1). Despite significant clinical and surgical improvements, the prognosis of GC patients, especially in late stages of the disease, is generally poor and remains a huge challenge (2). It is generally believed that the occurrence of GC is a multi-step, multi-factor changing process induced by multiple etiologies and genetics. Risk factors related to the development of GC include smoking, obesity, and excessive nitrate intake in food (3). In recent years, increasing evidence has shown that pathogen infection is also an important factor in the development of GC (4).

Human cytomegalovirus (HCMV), one of the β-herpesviruses with a wide range of cellular affinities, is a common and persistent pathogen affecting the world’s population (5). Many studies have shown that HCMV infection can be involved in tumorigenesis as a tumorigenic factor (6–8). The detection rate of HCMV DNA in many tumor tissues, such as colon cancer (9), glioma (10), breast cancer (11), and prostate cancer (12), is significantly higher than that in the normal adjacent tissues. HCMV expresses different viral genes in different infection states, thereby affecting the function of host cells. HCMV-encoded viral regulatory proteins can directly activate oncogenes, thereby promoting the transformation of normal infected cells into malignant phenotypes and enhancing tumor progression (13, 14). In addition, HCMV can significantly change the immunological characteristics of infected cells, and the tumor immune microenvironment, which are important factors in the development of tumors (11, 15). Our previous research found that HCMV infection was associated with GC, and the detection rate of HCMV DNA in GC tumor tissues was significantly higher than that in adjacent tissues (16, 17). Furthermore, serum HCMV-specific IgG and IgM antibodies were significantly higher in GC patients than in healthy controls (18).

HCMV has 208 open reading frames throughout the genome and contains both a long unique sequence (UL) and short unique sequence (US) (19, 20). US31 is located in the US region of HCMV and is a member of the US1 family (21). Our previous study found that US31 can significantly alter the immunological function of monocytes and macrophages and promote the polarization of M1 macrophages. Overexpression of US31 in systemic lupus erythematosus can cause an inflammatory response and thus participate in the development of this disorder (22). However, the role of US31 in the occurrence and development of GC has not been reported.

In this study, we evaluated the expression of the HCMV US31 protein in GC tumor tissues and explored its possible relationship with the clinicopathological characteristics and prognosis of GC patients. In addition, the correlation between US31 and immune characteristics of GC patients was analyzed to determine the effect of US31 on the immune microenvironment of GC. Furthermore, we investigated the effect of US31 on the proliferation, migration, and invasion of GC cell lines.

## Materials and Methods

### Patients and Tissue Samples

We collected 537 GC tissue samples retrospectively from December 2008 to July 2011 in the Second Affiliated Hospital of Wenzhou Medical University (Wenzhou, People’s Republic of China). All tissues obtained from surgical resection were diagnosed as GC according to the American Joint Committee on Cancer guideline (8th edition), fixed immediately with formalin, and embedded in paraffin. Clinical characteristics such as gender, age, tumor size, depth of invasion, lymph node metastasis, TNM stage, postoperative chemotherapy, and relapse were collected. This study was approved by the Ethics Committee of the Second Affiliated Hospital of Wenzhou Medical University. An informed consent form was signed by all patients participating in this study.

### Cell Culture and Construction of a Recombinant Plasmid Expressing US31

Two GC cell lines (BGC-823, and SGC-7901) were purchased from the cell bank of the Chinese Academy of Medical Sciences. All cells were cultured in Dulbecco’s modified Eagle’s medium (DMEM; Gibco) containing 10% fetal bovine serum (FBS; Gibco) at 37°C in a 5% CO_2_ atmosphere.

The DNA fragment of the US31 coding sequence tagged with hemagglutinin (HA), from the HCMV Merlin strain, was inserted into the pcDNA3.1(+) vector by ligating into the NedI/XhoI sites. The plasmid was sequenced to confirm successful construction. When cultured cells reached 60–70% confluence, the plasmid was transfected into the cells using Lipofectamine 2000 (Invitrogen) and incubated at 37°C according to the experimental manual. The expression and location of US31 was examined using an anti-HA-Tag antibody *via* western blot and immunofluorescence, respectively.

### RNA Isolation and Quantitative Real-Time Polymerase Chain Reaction

Total RNA was extracted from transfected cells at 24 and 48 h using TRIzol reagent (Invitrogen). cDNA was then synthesized according to the manufacturer’s protocol. Real-time polymerase chain reaction (RT-qPCR) was performed to examine mRNA expression levels using a SYBR^®^ Green PCR Kit (Qiagen). Then, the expression levels of genes in transfected cells relative to the control group were calculated by the comparative Ct method (2^−ΔΔCt^). Glyceraldehyde 3-phosphate dehydrogenase was used as an internal loading control. All primers used are listed in **Table 1**.

**Table 1:** The primer sequences for RT-qPCR.

Gene (Homo)	Sequence (5′–3′)	
IL-1β	Forward Primer	AGCTACGAATCTCCGACCAC
	Reverse Primer	CGTTATCCCATGTGTCGAAGAA
IL-6	Forward Primer	ACTCACCTCTTCAGAACGAATTG
	Reverse Primer	CCATCTTTGGAAGGTTCAGGTTG
IL-8	Forward Primer	CCTGATTTCTGCAGCTCTGTG
	Reverse Primer	CCAGACAGAGCTCTCTTCCAT
NF-κB2	Forward Primer	ATGGAGAGTTGCTACAACCCA
	Reverse Primer	CTGTTCCACGATCACCAGGTA
TNF-α	Forward Primer	CTGGGCAGGTCTACTTTGGG
	Reverse Primer	CTGGAGGCCCCAGTTTGAAT
COX-2	Forward Primer	TGTGACTGTACCCGGACTGG
	Reverse Primer	TGCACATTGTAAGTAGGTGGAC
iNOS	Forward Primer	ACCAGAGGACCCAGAGACAA
	Reverse Primer	CCTGGCCAGATGTTCCTCTA
ICAM-1	Forward Primer	TCACCTATGGCAACGACTCC
	Reverse Primer	CAGTGTCTCCTGGCTCTGGT
GAPDH	Forward Primer	CAGGGCTGCTTTTAACTCTGGTAA
	Reverse Primer	GGGTGGAATCATATTGGAACATGT

### RNA-Sequencing and Functional Enrichment Analysis of Differentially Expressed Genes

An RNA-sequencing (RNA-seq) analysis was performed in transfected cells at 24 and 48 h at the Beijing Genomics Institute to determine the effect of US31 overexpression on mRNA levels of GC cells. Differentially expressed genes (DEGs) of two groups were compared using the edgeR package. The significance criteria were defined as | log_2_(fold change) | > 1 and a p value < 0.05. To determine the function of these DEGs, a functional enrichment analysis, including Gene Ontology (GO) terms and Kyoto Encyclopedia of Genes and Genomes (KEGG) pathways, was performed using the Shanghai Bohao Biotechnology website (http://enrich.shbio.com/index/ga.asp).

### Immunoprecipitation and Liquid Chromatography With Tandem Mass Spectrometry Analysis

The proteins that interact with US31 were identified by immunoprecipitation (IP) using a Pierce Magnetic HA-Tag IP/CoIP Kit (Thermo Fisher Scientific Inc., USA) according to the manufacturer’s instructions. Briefly, SGC-7901 cells were transiently transfected with recombinant US31-HA plasmid and harvested 48 h post-transfection. Subsequently, supernatants containing US31-HA-tagged proteins were added to pre-washed magnetic beads and incubated for 1 h at 25°C. The immunoprecipitate was then boiled in 2× SDS loading buffer, subjected to SDS-PAGE, and visualized by silver staining. The eluted proteins were identified by liquid chromatography with tandem mass spectrometry (LC-MS/MS) (Novogene, Beijing, China).

### Western Blotting

Total proteins were harvested, and western blots were performed as described previously (23). Briefly, proteins were separated using 10% sodium dodecyl sulfate-polyacrylamide gel electrophoresis and transferred onto polyvinylidene difluoride membranes. After blocking with 5% non-fat milk at 24°C for 1 h, the membranes were incubated with primary antibodies targeting HA-Tag (diluted 1:1000, Cell Signaling Technology) and glyceraldehyde 3-phosphate dehydrogenase (diluted 1:1000, Proteintech) at 4°C overnight. After that, membranes were incubated with secondary horseradish peroxidase-conjugated antibodies (dilution 1:5000, Cell Signaling Technology) for 2 h at 25°C. Proteins were detected using a Bio-Rad Imaging System.

### Immunofluorescence

Cells were seeded on glass coverslips in 6-well plates, transfected with US31, and incubated at 37°C for 48 h. Next, the cells were fixed in 4% formaldehyde, permeabilized with 0.5% Triton X-100 in phosphate-buffered saline, blocked with 5% bovine serum albumin at 37°C for 30 min, and incubated with primary antibodies for HA-Tag (diluted 1:200) and US31 (diluted 1:1000) at 4°C overnight. Subsequently, cells were incubated with a secondary fluorescence-conjugated goat antibody and stained with 4′,6-diamidino-2-phenylindole. Finally, images were collected using a fluorescence microscope. The fluorescence signals were visualized as green (Alexa Fluor 488 HA-Tag) and red (Alexa Fluor 594 US31).

### Tissue Microarray and Immunohistochemistry

The tissue microarray (TMA) was constructed and immunohistochemistry (IHC) performed as described previously (23). Briefly, TMA slides were dewaxed in xylene, incubated in a 0.5% hydrogen peroxide bath for 10 min, blocked in sheep serum for 30 min, and then incubated with a US31 polyclonal rabbit antibody (diluted 1:500), or CD4, CD8, CD66b, and CD163 polyclonal rabbit antibodies (diluted 1:1000) at 24°C for 2 h. After incubation with secondary antibodies at 24°C, and staining with 3,3′-diaminobenzidine (Dako) and counterstaining with hematoxylin, the TMA slides were analyzed using an Easyscan6 digital slice scanner (MOTIC Medical Diagnostic Systems). All samples were evaluated independently by two pathologists without knowledge of the patients’ information.

The expression of US31 was rated 0 (negative), 1 (weak), 2 (medium), or 3 (strong) based on the color intensity. In addition, five high-power fields of each slice were selected randomly and the percentage of positive cells in each was recorded and scored as: 0 (< 5%), 1 (5–25%), 2 (26–50%), 3 (51–75%), and 4 (> 75%). The total US31 score was obtained by multiplying the color intensity and positive cell percentage scores. For the expression of CD4, CD8, CD66b, and CD163, the scores were normalized by dividing the number of positive cells by the total area (cells/mm^2^).

### Cell Proliferation Assays

We performed the cell counting kit-8 (CCK8) assay to evaluate GC cell proliferation. Briefly, transfected cells (5000 cells/well) were seeded into 96-well plates and cultured in 100 μl DMEM containing 10% FBS. Then, 10 μl CCK8 solution was added to each plate at 24, 48, and 72 h. After incubation at 37°C for 2 h, absorbance was measured at 450 nm in a microplate reader.

### Transwell Migration and Invasion Assays

Transwell migration and invasion assays were performed as described previously (23). Briefly, transfected cells were collected and counted. For the migration assay, 200 μl of DMEM containing 2 × 10^5^ cells was added to the upper chamber, and 500 μl of DMEM with 10% FBS was added into the lower chamber. For the invasion assay, the upper chamber was coated with 100 μl Matrigel (BD Pharmingen) diluted in serum-free DMEM (1:10) before cells were added. After incubation at 37°C for different time periods, cells were fixed with 4% paraformaldehyde for 15 min, stained with 0.1% crystal violet for 15 min, and counted.

### Statistical Analyses

Statistical analyses were performed with SPSS version 20.0 (IBM) and Prism 7.0 (GraphPad) software. Data are presented as means ± standard deviation or percentages. Comparisons between two groups were made using two-tailed Student’s t or χ^2^ tests. Survival was determined using the Kaplan-Meier method, and the log-rank test was applied for survival comparisons. *P < 0.05, **P < 0.01, and ***P < 0.001 indicated statistically significant differences.

## Results

### Clinicopathological Characteristics of Patients

A total of 573 GC patients, including 414 (72.3%) males and 159 (27.7%) females, were enrolled in the study to evaluate the expression of US31. The mean age of these patients was 58.9 ± 11.3 years (range, 20 to 86 years). The study also included TNM classification and pathologic staging of the patients. There were 277 (48.3%) stage I/II patients and 296 (51.7%) stage III/IV patients. Three hundred ninety-two (68.4%) patients received post-operative chemotherapy. Relapse occurred in 233 (40.7%) patients and 253 (44.2%) died with a median follow-up of 59 months (range, 0 to 101 months). Clinicopathological characteristics of all GC patients are shown in **Table 2**.

**Table 2:** Clinicopathological characteristics of gastric cancer patients and their correlation with the expression of HCMV US31 protein.

Variables	All patients(n = 573)	US31-positive expression(n = 504)	US31-negative expression(n = 69)	*P* value
Gender				0.078
Female	159 (27.7 %)	146 (29.0 %)	13 (18.8 %)	
Male	414 (72.3 %)	358 (71.0 %)	56 (81.2 %)	
Age				0.846
≤ 60	305 (54.0 %)	267 (53.8 %)	38 (55.1 %)	
> 60	260 (46.0 %)	229 (46.2 %)	31 (44.9 %)	
T stage				0.716
T1	126 (22.0 %)	112 (22.2 %)	14 (20.3 %)	
T2/T3/T4	447 (78.0 %)	392 (77.8 %)	55 (79.7 %)	
N stage				0.357
N0	220 (38.4 %)	197 (39.1 %)	23 (33.3 %)	
N1/N1/N2	353 (61.6 %)	307 (60.9 %)	46 (66.7 %)	
M stage				< 0.001^***^
M0	554 (96.7 %)	493 (97.8 %)	61 (88.4 %)	
M1	19 (3.3 %)	11 (2.2 %)	8 (11.6 %)	
Pathologic stage				0.169
I + II	277 (48.3 %)	249 (49.4 %)	28 (40.6 %)	
III + IV	296 (51.7 %)	255 (50.6 %)	41 (59.4 %)	
Tumor size				0.005^**^
≤ 4.5cm	346 (60.4%)	315 (62.5%)	31 (44.9%)	
>4.5cm	227 (39.6%)	189 (37.5%)	38 (55.1%)	
Chemotherapy				0.826
No	181 (32.1 %)	160 (31.7 %)	21 (30.4 %)	
Yes	392 (68.4 %)	344 (68.3 %)	48 (69.6 %)	
Relapse				0.120
No	340 (59.3 %)	305 (60.5 %)	35 (50.7 %)	
Yes	233 (40.7 %)	199 (39.5 %)	34 (49.3 %)	

**Statistically significant (P < 0.01). ***Statistically significant (P < 0.001).

### US31 Was a Prognostic Biomarker to Predict Tumor Size and Distant Metastasis

**Figure 1:**
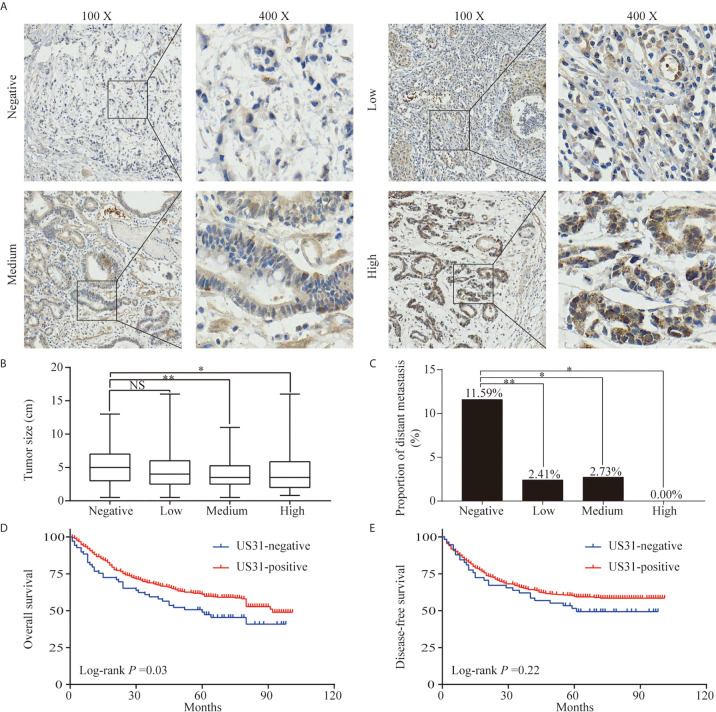
Expression of US31 in gastric cancer (GC) and its impact on prognosis. (**A**) Representative immunohistochemical staining of US31 in GC tissue. Brown indicates positive staining. **(B)** Correlation between the expression level of US31 and tumor size. **(C)** Correlation between the expression level of US31 and distant metastasis. **(D)** Kaplan-Meier curves for overall survival of GC patients with US31 expression. **(E)** Kaplan-Meier curves for disease-free survival of GC patients with US31 expression. *P < 0.05 and **P < 0.01. ns, no significance.

IHC showed that US31 was expressed mainly in the cytoplasm (**Figure 1A**). According to the different intensities by IHC, we divided these patients into US31-negative (n = 69) and US31-positive groups (n = 504). Correlations between the expression of US31 and the clinical characteristics of GC patients were analyzed (**Table 2**). The expression of US31 was significantly associated with tumor size (*P* = 0.005) and distant metastasis (*P* < 0.001). Correlations of US31 with gender, age, T stage, N stage, and postoperative chemotherapy were not statistically significant (*P* > 0.05).

US31-positive patients were clustered into high (n = 68), medium (n = 146), and low (n = 290) US31 expression groups. The results further showed that the tumor size and distant metastasis rate of GC were significantly negatively correlated with the expression of US31 (**Figures 1B, C** **)**. This indicated that the expression of US31 may inhibit the occurrence and development of GC. A Kaplan–Meier analysis suggested that US31-positive patients had better prognosis than US31-negative patients for overall survival (OS; log-rank *P* = 0.03; hazard ratio (HR) = 0.69; 95% confidence interval (CI) = 0.46–1.02) (**Figure 1D**), but not for disease-free survival (DFS; log-rank *P* = 0.22; HR = 0.79; 95% CI = 0.51–1.21) (**Figure 1E**). In the univariate analysis of OS, expression of US31 was a significantly independent prognostic biomarker (*P* = 0.03; HR = 0.69; 95% CI = 0.49–0.98). However, the multivariate analysis showed that expression of US31 was not an independent predictor for OS (*P* = 0.53; HR = 0.89; 95% CI = 0.62–1.28). **Table 3** shows detailed information of the univariate and multivariate analyses. Overall, higher US31 expression could inhibit GC progression and suggest a better prognosis.

**Table 3:** Univariate and multivariate Cox regression analysis of overall survival in patients with gastric cancer.

Variables	Univariate Cox analysis		Multivariate Cox analysis	
	HR (95% CI)	*P* value	HR (95% CI)	*P* value
Gender (male vs. female)	1.10(0.83-1.44)	0.508	1.12 (0.86-1.47)	0.405
Age (>60 vs. ≤60)	1.36(1.06-1.75)	0.015^*^	1.18(0.88-1.59)	0.269
TNM stage (III+IV vs. I+II)	4.91(3.63-6.64)	<0.001^***^	1.82(1.28-2.59)	<0.001^***^
Chemotherapy (Yes vs. No)	2.30(1.68-3.14)	<0.001^***^	1.13(0.80-1.61)	0.480
Relapse (Yes vs. No)	36.13(24.03-54.33)	<0.001^***^	35.79(21.31-60.10)	<0.001^***^
Tumor size (>4.5cm vs. ≤4.5cm)	3.28(2.55-4.22)	<0.001^***^	1.59(1.19-2.12)	<0.002^***^
US31 expression (positive vs. negative)	0.69(0.49-0.98)	0.037^*^	0.89(0.62-1.28)	0.534

Statistically significant (P < 0.001). *P < 0.05 and ***P < 0.001.

### US31 Inhibited the Proliferation, Migration, and Invasion of GC Cells

**Figure 2:**
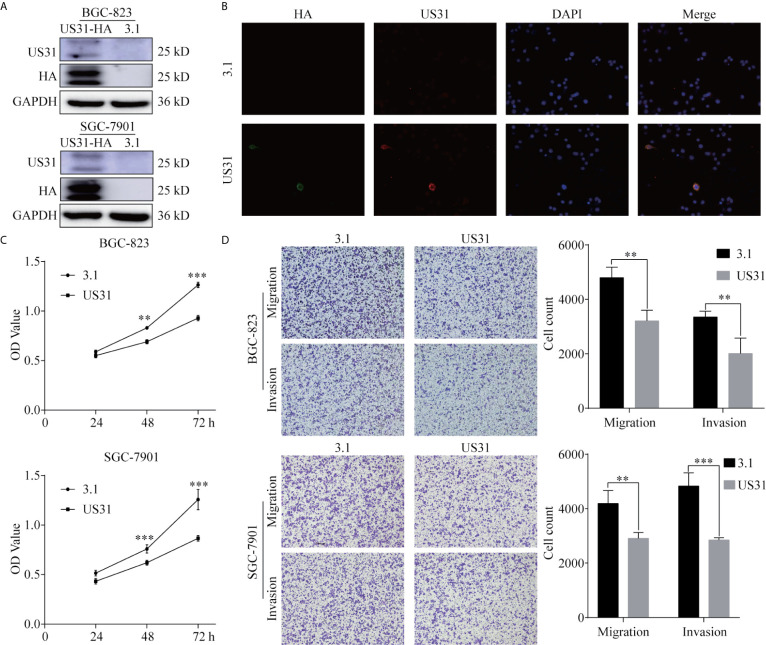
Overexpression of US31 significantly inhibits the proliferation, migration, and invasion of gastric cancer (GC) cells. **(A)** Overexpression of US31 in BGC-823 and SGC-7901 cells. **(B)** Immunofluorescence was used to detect the expression and location of US31 in GC cells. Green fluorescence represents the HA-tag; red fluorescence represents US31. US31 is mainly expressed in the cytoplasm and cell membrane. **(C)** The CCK-8 assay shows that US31 overexpression inhibits the proliferation of GC cells. **(D)** The Transwell assay shows that US31 overexpression inhibits the migration and invasion of GC cells. **P < 0.01 and ***P < 0.001. ns, no significance.

To investigate the effect of US31 on the biological function of GC cells, we transfected a plasmid into BGC-823 and SGC-7901 cells to overexpress US31. Overexpression was confirmed by western blot (**Figure 2A**). Results of the immunofluorescence assay showed that US31 was mainly expressed in the cytoplasm and cell membrane of GC cells (**Figure 2B**), which was consistent with the location shown in IHC. Then, we performed a CCK8 analysis to examine the effect of US31 on cell proliferation. Compared with the control group, US31 overexpression inhibited the proliferation of GC cells, especially BGC-823 cells (*P* < 0.05; **Figure 2C**). The Transwell migration and invasion assays showed that US31 overexpression inhibited cell migration and invasion of BGC-823 and SGC-7901 cells (*P* < 0.05; **Figure 2D**). Taken together, these results showed that US31 inhibited both the growth and migration of GC cells.

### High US31 Expression Was Associated With the Immune Microenvironment of GC

**Figure 3:**
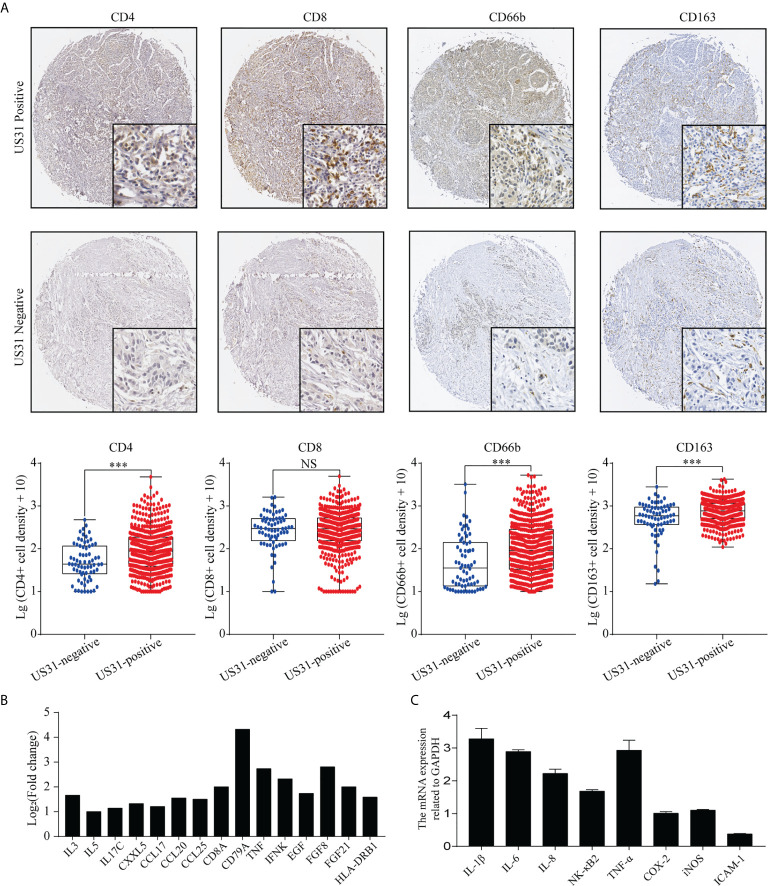
Association between US31 expression and various tumor-infiltrating immune cells and the immune response regulated by US31 expression of gastric cancer (GC) cells. **(A)** Representative immunohistochemical staining of tumor-infiltrating immune cells (CD4+, CD8+, CD66b and CD163 cells) in US31 positive and negative tissues. Overview picture is 100×; detailed picture is 400×. And the association between US31 expression and those tumor-infiltrating immune cells. **(B)** mRNA levels of representative immune-related genes measured by RNA-seq. **(C)** mRNA levels of representative immune-related genes in GC cells with US31 overexpression detected by real-time quantitative polymerase chain reaction. ***P < 0.001. ns, no significance.

HCMV infection can affect the tumor immune microenvironment. We analyzed the infiltration of CD4+, CD8+, CD66b+, and CD163+ cells into GC tissues to verify the effect of US31 expression on the immune microenvironment of GC. The results showed that the expression levels of CD4, CD66b, and CD163 in US31-positive tissues was significantly higher than those in US31-negative tissues (all *P* < 0.001; **Figure 3A**), while there was no significant difference in CD8 expression between the two groups (*P* = 0.98; **Figure 3A**).

**Figure 4:**
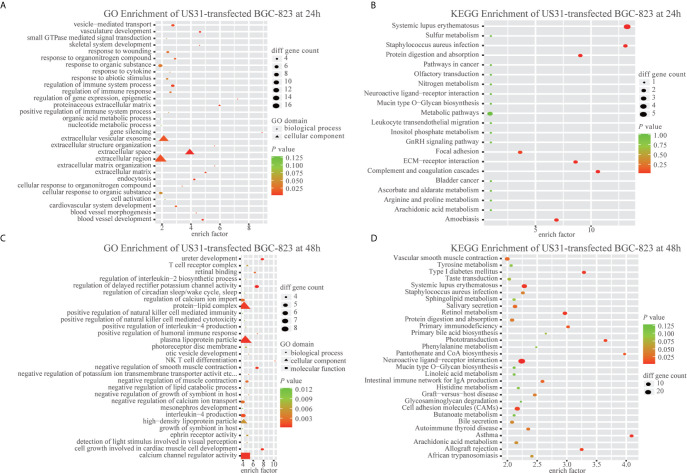
US31 expression regulates the immune response of gastric cancer (GC) cells. **(A–D)** Gene Ontology and KEGG enrichment analyses of differentially expressed genes caused by overexpression of US31 in BGC-823 at 24 and 48 h, respectively.

To study the effect of US31 overexpression on GC, RNA-seq was performed in transfected cells at 24 and 48 h. Based on the significance criteria (| log_2_(fold change) | > 1 and p value < 0.05), we obtained 51 and 1082 DEGs at 24 and 48 h, respectively. The mRNA levels of representative immune-related genes showed a significant increase using both RNA-seq and RT-qPCR detection after transfection of US31 for 48 h (**Figures 3B, C** **)**. In addition, GO and KEGG enrichment analysis suggested that overexpression of US31 activated the immune response and promoted the secretion of cytokines in GC (**Figure 4**).

To further investigate US31’s potential partners, we transfected the US31-HA plasmid into SGC-7901 cells and performed IP to obtain the proteins that potentially interacted with US31 (Additional file: **Supplementary Figure S1A**). Subsequently, LC-MS/MS analysis was performed to identify those proteins (Additional file: **Supplementary Table S1** and **S2**). As shown in Fig. S1, 824 proteins in total were shown to interact with US31, and NF-κB2 were in the list of those proteins. Then we used the gene list of those proteins for GO pathway analysis. Consistent with the results of RNA-seq, GO pathway analysis showed that the US31 partner proteins were the most enriched in cell-cell adhesion, NF-κB signaling, and the tumor necrosis factor (TNF)-mediated signaling pathway, among others (**Supplementary Table S3**). Taken together, these results implied that US31 may inhibit the progression of GC through mediating immune microenvironment.

## Discussion

Despite the rapid development of various precision therapies, the cause of GC is still poorly understood. Increasing evidence has suggested that infection with the oncogenic HCMV virus is associated with the progression of GC. However, the role of the expression of many HCMV genes in tumors is still unknown. Understanding the relationship between these genes and GC will help improve the treatment and prognosis of patients. In this study, we show, for the first time, that high expression of HCMV US31 can significantly improve the prognosis of GC patients by inhibiting the proliferation and metastasis of GC. Moreover, US31 can activate the host’s immune response and cytokine secretion. These results suggest that US31 might act as a better prognostic factor in the development of GC.

HCMV infection is closely related to the occurrence and progression of various tumors, including glioma, breast cancer, colorectal cancer, and prostate cancer. In addition, the diverse HCMV genes in human cancers present different functional phenotypes in tumors. For example, Lv et al. (24) reported that HCMV infection was associated with an increased gastrointestinal cancer risk. HCMV has a higher prevalence in patients with primary breast cancer and colorectal cancer with brain metastases, and the high expression of HCMV- immediate-early proteins (IE) in the tumor tissues of the patients suggests a poor prognosis (25). HCMV IE1 and IE2 can activate phosphoinositide 3-kinase/Akt signaling pathways, use oxidative phosphorylation to inhibit Rb protein function, and reduce the expression of p53 family proteins in gliomas (26). The US28 protein can promote the proliferation of tumor cells and increase the expression of cyclin D1 (27). In contrast, Herbein et al. (28) reviewed the tumor suppressor effects of HCMV and thought that HCMV may act as an oncolytic virus. HCMV can induce tumor cell apoptosis, regulate the tumor microenvironment, stimulate the infiltrating immune cells to kill the tumor cells, and thus act as a protective factor in the tumor. Therefore, we speculate that there existed different expression patterns of HCMV genes in tumor tissues, and these different patterns may have opposite effects in tumor progression. Our previous research showed that the UL133–UL138 locus, located in the ULb′ region of HCMV, may be crucial for the development of GC. The UL138 gene is a virus-associated tumor suppressor gene that interacts with HSP70 to inhibit proliferation and promote apoptosis of GC cells. Here, we had demonstrated another tumor suppressor gene of HCMV that is US31, could predict a better prognosis of GC.

**Figure 5:**
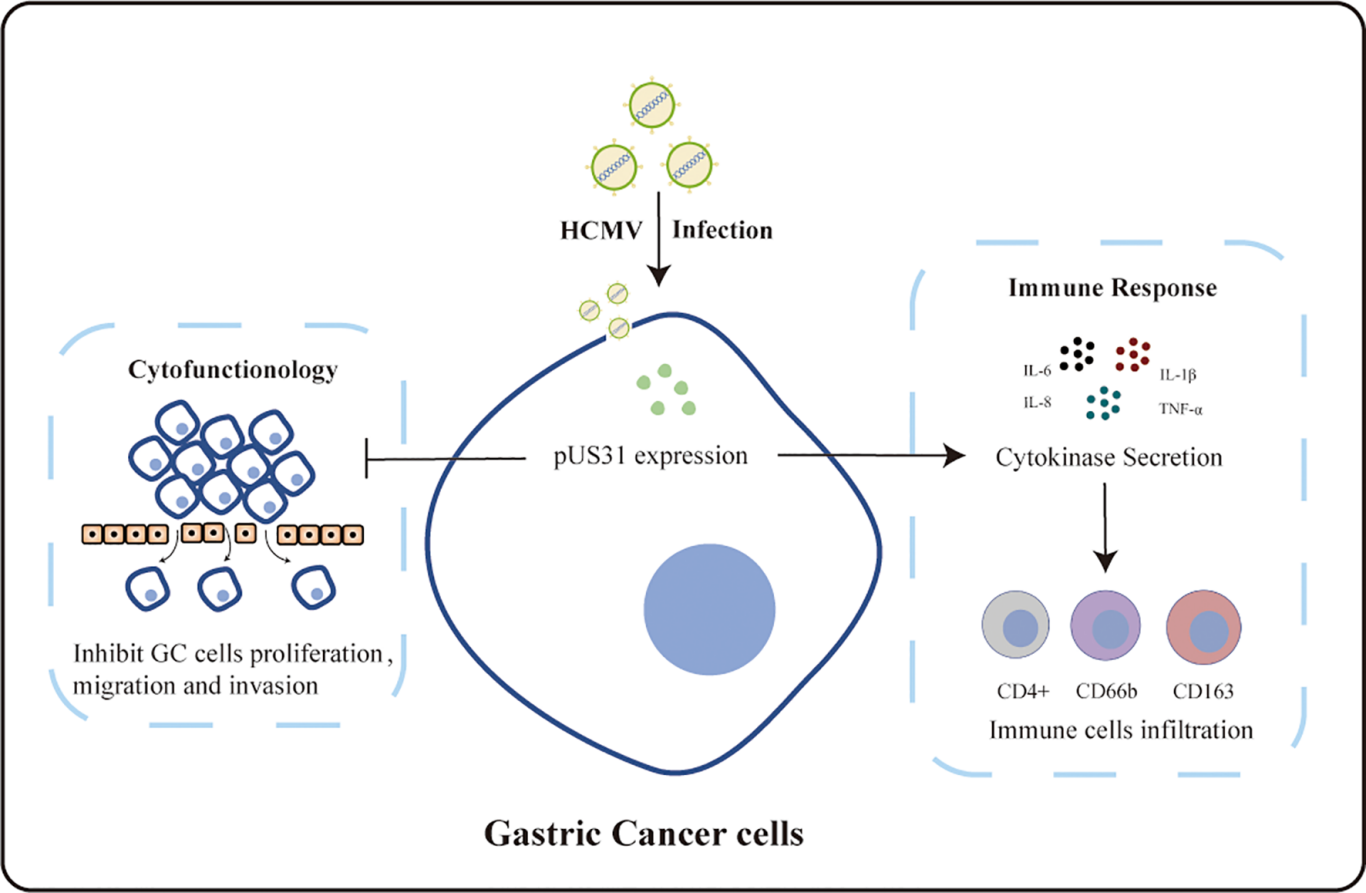
Proposed model for the function landscape of the HCMV-induced US31 expression in GCs.

The HCMV US1 family is mainly composed of US1, US31, and US32 genes. The US1 gene is an immediate-early gene, and the US32 gene is associated with the latent virus (29). However, there are few reports about the expression of US31 in HCMV infection and its effect in GC patients. In this study, we used a self-made specific affinity purified US31 antibody to analyze the expression of US31 in 573 GC tissues by IHC. The results showed that US31 was mainly expressed around the cytoplasm and cell membrane. US31 expression significantly correlated with tumor diameter and distant metastasis. Moreover, US31 protein overexpression was significantly associated with better prognosis of OS in GC patients. Consistent with these results, overexpression of US31 significantly inhibited the proliferation, migration, and invasion of GC cells *in vitro*. Taken together, US31 may be a potential prognostic biomarker to inhibit the development of GC (**Figure 5**).

Accumulating studies have demonstrated that the tumor microenvironment has a clear relationship with the prognosis of cancer patients. Alterations of the tumor microenvironment may result in the secretion of cytokines and activate various cell signaling pathways, thereby promoting tumor development. Tumor-infiltrating immune cells, including tumor-associated macrophages, dendritic cells, and lymphocytes, are the major components of the tumor microenvironment and important factors involved in the development of tumors (30).

Persistent infection of HCMV can stimulate the immune response of patients. This, in turn, leads to chronic inflammation and poor survival. HCMV can encode a human interleukin (IL)-10 homolog to suppress host immunity. Furthermore, HCMV-infected cells can secrete a variety of cytokines (such as IL-10 and transforming growth factor-β), extracellular matrix proteins, and angiogenic factors to change the tumor microenvironment (31). Chang et al. (32) found that there was a certain IL-10 homolog in the supernatant of HCMV-infected cells. After treatment with this supernatant, the maturation ability and ability to produce inflammatory cytokines of dendritic cells were significantly inhibited. In the current study, we found that the expression of US31 significantly correlated with the expression of CD4, CD66B, and CD163. Huang et al. (33) detected the infiltration of CD66b+ and CD163+ cells in 662 GC tissues by IHC. These results suggested that infiltration of CD66b+ neutrophils was associated with favorable tumor characteristics, while high infiltration of CD163+ tumor-associated macrophages was associated with disease progression and poor survival. Moreover, Jiang et al. (34) indicated that the Immune Score, a novel signature based on the expression of CD3, CD8, CD45, and CD66b in 251 GC tissues, could predict recurrence and survival of GC. Therefore, we speculate that the expression of US31 in GC may regulate the immune microenvironment of the tumor.

Based on the immune microenvironment, several inflammatory cytokines could activate the immune response and modulate an antitumoral response in the tumor. For example, TNF-α is an inflammatory mediator and functions as an anti-tumorigenic factor at high concentrations (35). IL-1β is also reported to mediate anti-tumor responses through T lymphocytes (36, 37). Our previous studies found that US31 interacted with NF-κB2 (22), leading to phosphorylated P100 polyubiquitination and activating NF-κB2 (22). This, in turn, induced monocyte- and macrophage-mediated inflammation and stimulated the differentiation of macrophages into M1 macrophages. Consequently, the NF-κB2 regulators and signaling molecules TNF-α, IL-8, CCL2, ICAM-1, and RelB were significantly upregulated. Consistent with previous studies, our current results showed that the expression of US31 could also upregulate the secretion of inflammatory cytokines such as TNF-α, IL-1β, and IL-6 in GC cells. Furthermore, GO pathway analysis showed that the proteins that interact with US31 identified by MS were mainly involved in immune- and inflammation- related pathways. These results suggest that US31 may act as an immune- and inflammation-related factor in GC, thereby inhibiting the progression of cancer. Nevertheless, how US31 regulates those cytokines and GC progression is unclear and requires further in-depth study.

In summary, this study reports, for the first time, that the HCMV US31 gene is a potential prognostic factor for GC patients than those in current use. Its expression in GC tissue was significantly associated with tumor size and distant metastasis. Moreover, the overexpression of US31 can inhibit the proliferation, migration, and invasion of GC cells. In addition, US31 can activate the immune response and regulate the tumor immune microenvironment. Although the mechanism underlying the effect of US31 on the immune microenvironment of GC requires further research, this study provides new ideas for the treatment and prediction of the prognosis of GC patients.

## Data Availability Statement

The original contributions presented in the study are included in the article/**Supplementary Material**.. Further inquiries can be directed to the corresponding authors.

## Ethics Statement

This study was approved by the Review Board of the Second Affiliated Hospital of Wenzhou Medical University, protocol number: LCKY2020-130.

## Author Contributions

SY, YH and CC carried out experiments and drafted the manuscript. SC and XT performed the experiments and statistical analysis. XWS, XC and XYS acquired the data and material support. RG, WX, GG and DX analyzed and interpreted the data. HZ, BD and XH revised the manuscript and finally approved the version of the manuscript for publication. SS, XX and XS designed experiments and provided the project funding, revised the manuscript and finally approved the version of the manuscript for publication. All authors contributed to the article and approved the submitted version.

## Funding

This study was supported by grants from the National Nature Science Foundation of China (Grant Nos.: 32070151), the Key R&D Program of Zhejiang Province (Grant No.: 2020C03029), and the Zhejiang Provincial Natural Science Foundation of China (Grant No. WKJ-ZJ-1806).

## Conflict of Interest

The authors declare that the research was conducted in the absence of any commercial or financial relationships that could be construed as a potential conflict of interest.

## Supplementary Material

Supplementary Figure 1 Immunoprecipitation and liquid chromatography with tandem mass spectrometry analysis results of US31 expression in gastric cancer (GC) cells. **(A)** The US31 was immunoprecipitated by HA-tag. **(B)** Silver staining of US31 and its partner proteins. The US31 was covered by the light IgG. **(C)** Venn diagram showing 824 proteins were identified to interact with US31. NF-κB2 was in the list. **(D)** US31 interacting proteins identified by IP-MS were clustered by GO molecular function.

Supplementary Table 2 The US31 interacting proteins list by LC-MS/MS.

Supplementary Table 3 Enrichment list of US31 interacting proteins in GO.
