# Erratum: The Human Cytomegalovirus US31 Gene Predicts Favorable Survival and Regulates the Tumor Microenvironment in Gastric Cancer

**DOI:** 10.3389/fonc.2021.715746

**Published:** 2021-06-17

**Authors:** Frontiers Production Office

**Affiliations:** Frontiers Media SA, Lausanne, Switzerland

**Keywords:** human cytomegalovirus, gastric cancer, US31, tumor immune microenvironment, prognostic factor

Due to a production error, an author name was incorrectly spelled as “Xiadong Chen”. The correct spelling is “Xiaodong Chen”. 

The publisher apologizes for this mistake. The original version of this article has been updated.

