# Supplementary figures and images for: The Human Cytomegalovirus US31 Gene Predicts Favorable Survival and Regulates the Tumor Microenvironment in Gastric Cancer
Source: Front Oncol. 2021 Apr 20;11:614925. doi: 10.3389/fonc.2021.614925 (PMC8093799; doi:10.3389/fonc.2021.614925)

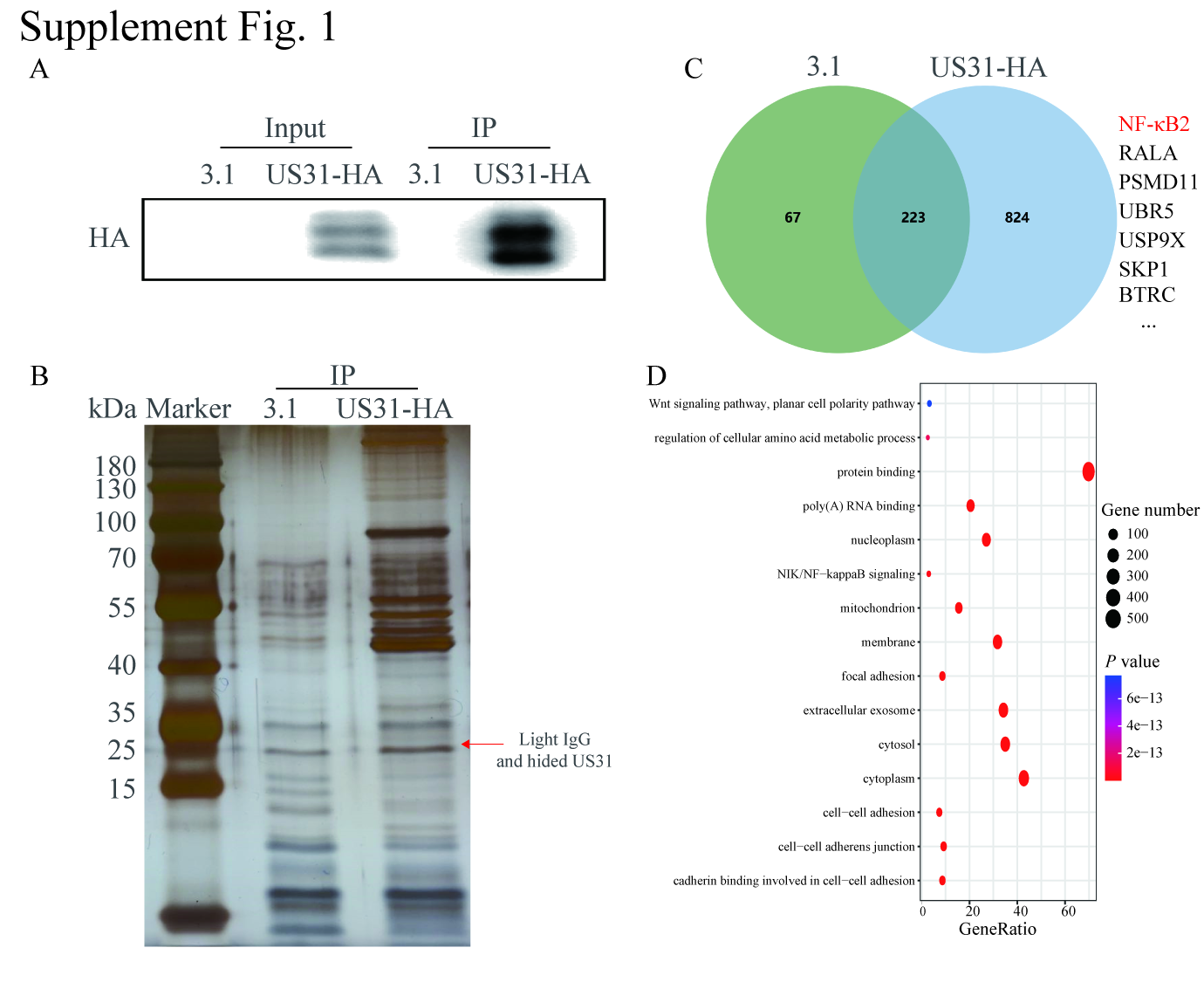

Supplement: Supplementary Figure 1 — Immunoprecipitation and liquid chromatography with tandem mass spectrometry analysis results of US31 expression in gastric cancer (GC) cells. (A) The US31 was immunoprecipitated by HA-tag. (B) Silver staining of US31 and its partner proteins. The US31 was covered by the light IgG. (C) Venn diagram showing 824 proteins were identified to interact with US31. NF-κB2 was in the list. (D) US31 interacting proteins identified by IP-MS were clustered by GO molecular function. [file Image_1.tif]

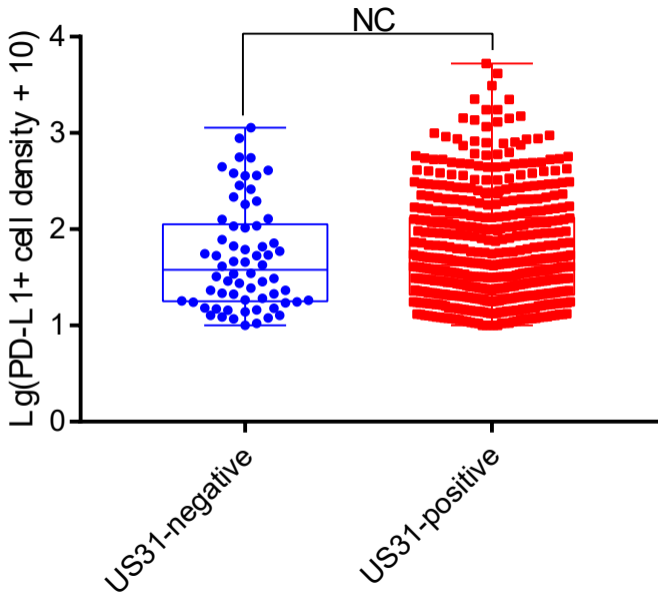

Supplement: Supplementary file 5 [file DataSheet_1.pdf]
