# Supplementary material for: The Human Cytomegalovirus US31 Gene Predicts Favorable Survival and Regulates the Tumor Microenvironment in Gastric Cancer
Source: Front Oncol. 2021 Apr 20;11:614925. doi: 10.3389/fonc.2021.614925 (PMC8093799; doi:10.3389/fonc.2021.614925)
Supplement: Supplementary file 4 [file Table_1.docx]

Table S1 Ten of 824 differential proteins interacted with US31 by LC-MS/MS.

| Protein list | Description |
| --- | --- |
| NF-κB2 | NFKB2_HUMAN Nuclear factor NF-kappa-B p100 |
| RALA | Ras-related protein Ral-A |
| PSMD11 | 26S proteasome non-ATPase regulatory subunit 11 |
| PSMD12 | 26S proteasome non-ATPase regulatory subunit 12 |
| UBA52 | Ubiquitin-60S ribosomal protein L40 |
| UBR5 | E3 ubiquitin-protein ligase UBR5 |
| STUB1 | E3 ubiquitin-protein ligase CHIP |
| USP9X | Probable ubiquitin carboxyl-terminal hydrolase FAF |
| SKP1 | S-phase kinase-associated protein 1 |
| BTRC | Beta-transducin repeat containing isoform 3 |
